# Thermally tolerant intertidal triplefin fish (Tripterygiidae) sustain ATP dynamics better than subtidal species under acute heat stress

**DOI:** 10.1038/s41598-021-90575-y

**Published:** 2021-05-26

**Authors:** Jaime R. Willis, Anthony J. R. Hickey, Jules B. L. Devaux

**Affiliations:** School of Biological Sciences, The University of Auckland, Auckland, 1142 New Zealand

**Keywords:** Physiology, Metabolism, Ecology, Ecophysiology

## Abstract

Temperature is a key factor that affects all levels of organization. Minute shifts away from thermal optima result in detrimental effects that impact growth, reproduction and survival. Metabolic rates of ectotherms are especially sensitive to temperature and for organisms exposed to high acute temperature changes, in particular intertidal species, energetic processes are often negatively impacted. Previous investigations exploring acute heat stress have implicated cardiac mitochondrial function in determining thermal tolerance. The brain, however, is by weight, one of the most metabolically active and arguably the most temperature sensitive organ. It is essentially aerobic and entirely reliant on oxidative phosphorylation to meet energetic demands, and as temperatures rise, mitochondria become less efficient at synthesising the amount of ATP required to meet the increasing demands. This leads to an energetic crisis. Here we used brain homogenate of three closely related triplefin fish species (*Bellapiscis medius, Forsterygion lapillum,* and *Forsterygion varium*) and measured respiration and ATP dynamics at three temperatures (15, 25 and 30 °C). We found that the intertidal *B. medius* and *F. lapillum* were able to maintain rates of ATP production above rates of ATP hydrolysis at high temperatures, compared to the subtidal *F. varium*, which showed no difference in rates at 30 °C. These results showed that brain mitochondria became less efficient at temperatures below their respective species thermal limits, and that energetic surplus of ATP synthesis over hydrolysis narrows. In subtidal species synthesis matches hydrolysis, leaving no scope to elevate ATP supply.

## Introduction

Temperature exerts a profound effect on all levels of organization; with no single organism, capable of withstanding the full spectrum of temperatures across the biosphere^1,2^. This is true of ectotherms, which lack active thermoregulatory processes and especially true for intertidal species which experience large fluctuations in ambient temperature over the course of a day^1^. With anthropogenic climate change shifting mean and extreme temperature patterns, understanding the basis of thermal tolerance and thermal adaptation is essential^3–5^. Temperature typically promotes a near exponential rise in metabolic rate until a critical threshold is reached, which is then followed by a sharp decline^6^. What ultimately mediates this metabolic collapse at high temperature remains controversial, and oxygen limitation has been proposed to underpin thermal tolerance in aquatic ectotherms^7,8^. However, this concept is debated, as underlying literature finds support for and against its application^8–12^.

Perhaps the most central role of metabolism is to maintain a tight balance between ATP production and consumption. In almost all animals that live independent from a host, mitochondria are the predominant source of ATP, and oxidative phosphorylation (OXPHOS) provides ~ 90% of the cellular ATP, around 15 times more than that generated via fermentative glycolysis^13,14^. Considerable work has focused on the mitochondrion’s role, specifically cardiac mitochondria, as the linchpin for thermal tolerance^15^. With rising temperature, the fraction of oxygen (O_2_) flux and carbon substrate consumed by mitochondria escalates, yet the energy released is increasingly directed toward an apparent futile cycle, or “leak” of protons across the inner mitochondrial membrane^14–16^.

Rising temperatures increase cellular ATP demands^6^, yet mitochondrial efficiency and outright ATP production may decline, even with saturating O_2_^15,17^. In sum, a mismatch between an organism's capacity to meet increasing ATP demands must occur. While considerable focus has been placed on respiration and ATP production, few have considered how these relate to rates of ATP hydrolysis at high ambient temperatures. This relatively simplistic concept of balance in cellular ATP-economics has yet to be followed in the contexts of temperature. We have the ability to measure ATP production as well as a simplistic measure of ATP hydrolysis under variable thermal stresses. This allowed us to generate a clearer picture with regard to energetic economy at high temperatures.

The role of mitochondria in thermal tolerance has mainly been assessed in liver and cardiac tissues, and hearts have been proposed to fail first, often with minimal increase in temperature^15,18,19^. However, nervous tissue is also excitable and may respond similarly with minor temperature fluctuations, and result in catastrophic loss of brain function^20–22^. The brain is also highly aerobic, as an excitable tissue with high sustained energetic demands^23–27^. Moreover, the resting brain is the greatest energy consumer in vertebrates, and even within ectotherms the brain may use upwards of 20% of resting O_2_ demands^28^. Action potentials and consequent restoration of ion balance consumes up to 80% of total ATP in active neurons and these pathways, along with synaptic transmission, are thermally sensitive^28,29^.

**Figure 1:**
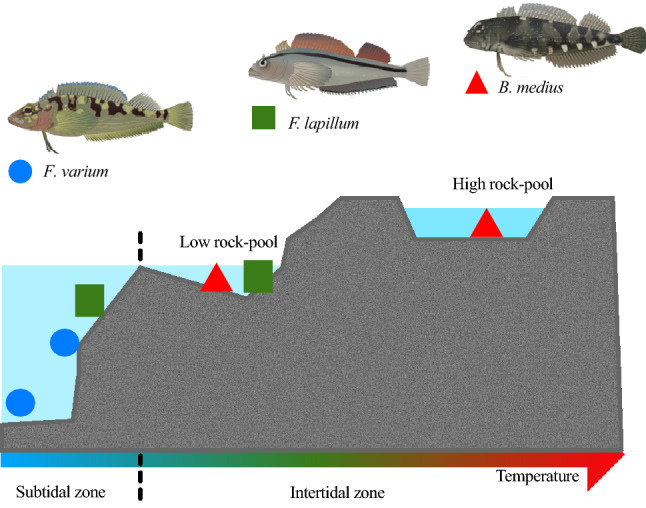
Triplefin species used in this study and their respective distributions. *Bellapiscis medius* is a high intertidal species that experiences a wide range of temperatures daily. *Forsterygion lapillum* is a shallow subtidal species that occurs at depths around 5–10 m and experiences a narrower temperature window than *B. medius*. *Forsterygion varium* is a deeper sub-tidal species found at depths of 8–20 m and experiences the narrowest temperature range of all three species. Temperatures in these rock pools vary with the tides and can peak in the summer months at ~ 30 °C (McArley et al., 2018; 2019). Triplefin images courtesy of Vivian Ward & Kendall Clements.

Intertidal marine ectotherms typically experience large diurnal and tidally mediated temperature fluctuations, relative to subtidal species, and consequently show physiological adaptations to survive thermal stress^31^. Adaptations can be detected through contrasting related species of distinct ecotypes^32^, and here we employ the New Zealand triplefin fish (Family: Tripterygiidae) model. We tested three species*, Bellapiscis medius, Forsterygion lapillum* and *Forsterygion varium* that respectively occupy niches within the high (0–5 m) and mid (1–10 m) intertidal zones and subtidal zones (4–15 m) and show distinct critical thermal maxima (CT_max_) that reflect their thermal habitats (Fig. 1)^33,34^. Earlier work on triplefin species has shown clear differences in heart mitochondrial efficiency and stability at high temperatures, with the intertidal *B. medius* maintaining the lowest leak respiration rates compared with the subtidal *F. varium* and *F. malcolmi*^33^*.* The aim of the present study was to investigate the effects of temperature on the ATP dynamics and overall mitochondrial function of three spatially diverse triplefin species. We hypothesised that mitochondrial efficiency would decrease as ambient temperature increased, and more specifically, that overall energetic balance would become closely matched as ATP supply is diminished and is unable to meet the ever-increasing demand. As such, we expect the rockpool exclusive *B. medius* to have the greatest mitochondrial efficiency at high temperatures, with sustained rates of ATP production above ATP demands, compared to the low intertidal *F. lapillum* and subtidal *F. varium*. Therefore, this work will provide a greater understanding into the underlying factors of thermal tolerance.

## Materials and methods

### Ethics

The research described conforms to the recommendations of the New Zealand Animal Welfare Act of 1999. All experiments were conducted under the approval of the animal ethics committee and animal welfare officer of the University of Auckland, New Zealand (permit R001551). Study was undertaken in compliance with ARRIVE guidelines.

### Animals and housing

The rock-pool exclusive and high intertidal species (*Bellapiscis medius and Forsterygion lapillum,* respectively) were caught using bait traps and hand nets in rock pools and off piers. Subtidal species *Forsterygion varium* were caught by SCUBA. All fish were collected around the greater Auckland region (− 36.081588, 174.598804). Fish were then transported to the facilities of the University of Auckland and held in 30 L aerated tanks with recirculating seawater at 18 ± 0.5 °C, 200 µm filtered, and 35 ± 1 ppt salinity. Fish were checked daily and fed ad libitum every 3 days with raw prawn meat. After at least a week of acclimation, fish were euthanized for experimentation. The three species were chosen for their differing habitat depth and thermal histories. *B. medius* is found at a depth range of 0–2 m and has a CT_max_ of 31.9 °C ± 0.01^34^, *F. lapillum* is found at depths of 0–10 m and has a CT_max_ of 31.3 °C ± 0.07, while *F. varium* is found at depths of 0–30 m. The CT_max_ values for *F. varium* are not available, but this species does not routinely encounter water temperatures as high as 25 °C^33,34^.

### Tissue homogenization

Fish were euthanized via pithing of the spinal cord. The brain case was then removed and the brain was rapidly excised, weighed and placed into a marine fish specific ice-cold respiration buffer (MiR05; modified from Gnaiger, 2007; EGTA 0.5 mM, Lactobionic acid 60 mM, Taurine 20 mM, KH_2_PO_4_ 10 mM, HEPES 20 mM, d-Sucrose 160 mM, BSA 1 g/L, pH 7.1 at 30 °C) at a 1:10 weight to volume dilution. Brain bundles were gently homogenized via suction and expulsion through a 10 ml homogenising syringe and two decreasing gauge needles (16 and 25).

### Respirometry

**Figure 2:**
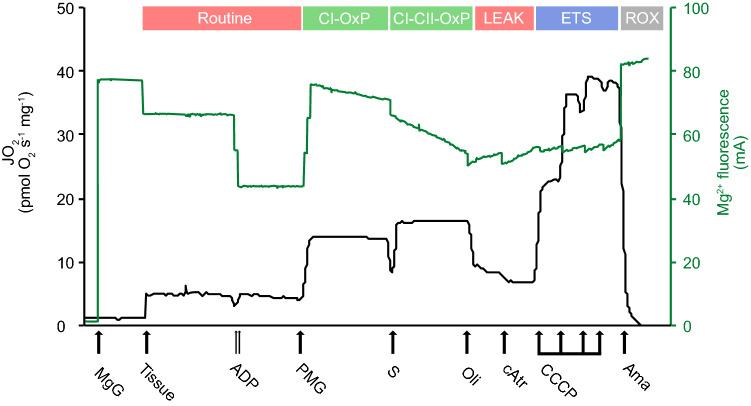
Representative trace of mitochondrial respiration assay from triplefin brain homogenates. Mitochondrial flux (pmol O_2_s^−1^ mg^−1^) on the left axis and Mg^2+^ fluorescence (mA) on the right axis. Addition of tissue and titration of mitochondrial substrates and inhibitors at saturated concentrations are indicated by the arrows and follow the SUIT protocol outlined above. *ADP* Adenosine diphosphate, *P* Pyruvate, *M* Malate, *G* Glutamate, *S* Succinate, *Oli* Oligomycin, *cAtr* Carboxyatractyloside, *CCCP* Carbonyl cyanide m-chloro phenyl hydrazone, *Ama* Antimycin A. Different mitochondrial states are represented above the figure.

Mitochondrial respiration assays were performed using Oroboros Oxygraph-2k respirometers (Oroboros instruments, Innsbruck, Austria). The oxygen standard was equilibrated to air levels according to chamber temperature (~ 290, 255 and 217 μM O_2_ at 15, 25 and 30 °C respectively) prior to experiments. Weight specific mitochondrial respiration flux (JO_2_) was calculated in real time using the negative time derivative of the O_2_ concentration and expressed as pmol O_2_ s^−1^ mg^−1^. Two assay protocols were used to track respiration as well as ATP dynamics at three temperatures (Fig. 2). Assay temperatures in this study represented the average seasonal habitat temperature, standard temperature and maximal temperature reached in rock-pools in summer (i.e., 15, 25 and 30 °C, respectively)^34^. A substrate-uncoupler-inhibitor-titration assay (SUIT) was used to test (i) mitochondrial components of the electron transport system (ETS) in a stepwise fashion, measured with the maximum O_2_ flux mediated by each titration, (ii) ATP production through OXPHOS and (iii) ATP dynamics of triplefin brain mitochondria (Fig. 2). Both assays differed by the addition of either ADP or ATP. Brain homogenate (~ 10 mg wet weight equivalent) was distributed equally between two parallel respirometry chambers and left until steady-state respiration was reached. Then, 2 mM final concentration of ADP (assay 1, one chamber) or ATP (assay 2, second chamber) was added to measure respiration rates supported by endogenous substrates (Routine state). The addition of ADP primed OXPHOS, while the addition of ATP forced hydrolysis rates. The NADH_2_-generating substrates pyruvate (5 mM), malate (2 mM), and glutamate (10 mM) were then added to stimulate CI-OXPHOS. Subsequently, succinate (10 mM) was added to stimulate complex II (CII) and allow the measurement of OXPHOS with the combined inputs of CI and CII (CI&CII-OXPHOS). Respiration attributed to proton leak (LEAK) was assessed with the addition of the ATP_F0–F1_ synthase inhibitor oligomycin (2.5 μM). The contribution of adenine nucleotide translocator (ANT) to LEAK was calculated as the difference prior and after the addition of the ANT inhibitor carboxyatractyloside (cAtr; 5 μM). Mitochondria were then uncoupled from OXPHOS with repeated titrations of carbonyl cyanide m-chloro phenyl hydrazone (CCCP; 0.5 μM titration steps) to determine the maximum ETS capacity without the limitation of the phosphorylating system (ATP_F0–F1_ and ANT). Finally, non-mitochondrial respiration was determined by the addition of the complex III inhibitor antimycin A (Ama; 2.5 μM) was titrated to inhibit respiration (Fig. 2).

### ATP dynamics: assay and kinetics

Standard respiration media (MiR05) does not contain calcium (Ca^2+^) or sodium (Na^+^). As Na^+^ and Ca^2+^ salts are required to activate cellular ATPases; NaCl (5 mM) and CaCl_2_ (0.25 mM) were added before homogenate to reach maximum ATP hydrolysis rates. Maximum OXPHOS rates cannot be achieved in the absence of Mg^2+^, which is required for proper cellular function and stabilisation of ATP and ADP^35^. ATP and ADP kinetics were measured fluorometrically, as described elsewhere^36–39^. Briefly, as both ADP and ATP require Mg^2+^ for stabilisation, free Mg^2+^ [Mg^2+^] _free_ within the O2K chamber was monitored using Magnesium-Green™ (5 mM). The fluorescent signal (470/530 nm; Ex/Em) was calibrated by two subsequent titrations of MgCl_2_ (1.25 mM each) so that (i) Mg^2+^ dependent reactions can be achieved and (ii) Mg^2+^-free ADP and ATP are stabilised. As binding varies with temperature, independent assays were run at each experimental temperature without sample to determine binding kinetics between ADP-Mg^2+^, ATP-Mg^2+^ and Mg^2+^-MgG. Post fluorescent signal calibration, ADP and ATP (Mg^2+^-free) were titrated stepwise (1.25–2.5–3.75–5–6 mM) (Supplementary Fig. S1). This allowed the construction of ADP and ATP binding curves to Mg^2+^. The ratio of the slopes between the two curves at working ATP/ADP concentrations for assays 1 and 2 were used to determine a fluorescence correction factor for each assay at each experimental temperature (Supplementary Fig. S1). The experimental ADP signals were subsequently multiplied by the correction factor to determine rates of net ATP production^36,40^. ATP hydrolysis rates were determined during the SUIT assay protocol following the addition of Oligomycin to inhibit the ATP synthase. Using this rate, we could calculate the overall ATP production rate through the sum of the net ATP production rate with the ATP hydrolysis rate. Overall production and hydrolysis rates provide insights into the condition of the tissue, but more physiologically relevant information is acquired from P/O ratios; the amount of ATP produced per molecule of oxygen consumed. Changes in this ratio across temperature are calculated by dividing the rate of overall ATP production by O_2_ consumption during CI&CII-OXPHOS.

### Calculations and statistical analyses

Respiration data is presented as mean (n = 10 individuals) ± s.e.m (alternatives are otherwise stated). Respiratory control ratios (RCR) are calculated as (State 3-State 4)/State 3. State 3 corresponds to a more recently defined OXPHOS state when mitochondria are exposed to sufficient substrates and ADP, whereas state 4, or LEAK, is measured in the absence of ADP, or also in the presence of oligomycin (state 4_o_). In this present study on homogenates RCRs were calculated as (CI&CII-OXPHOS-LEAK)/CI&CII-OXPHOS (e.g., (State 3-State 4_o_)/State 3). The reserve respiratory capacity is calculated as CI&CII-OXPHOS—ETS. Prism (Vers.8) was used to conduct independent *t-test* between mitochondrial states when homogeneity of variance was verified. Two-way ANOVAs were performed to analyse the effect of temperature and species on respiration rates and ATP dynamics with a Greenhouse–Geisser correction when sphericity was not assumed. Tukey-Post Hoc tests were performed for pairwise comparison.

## Results

### Respiration assays

**Figure 3:**
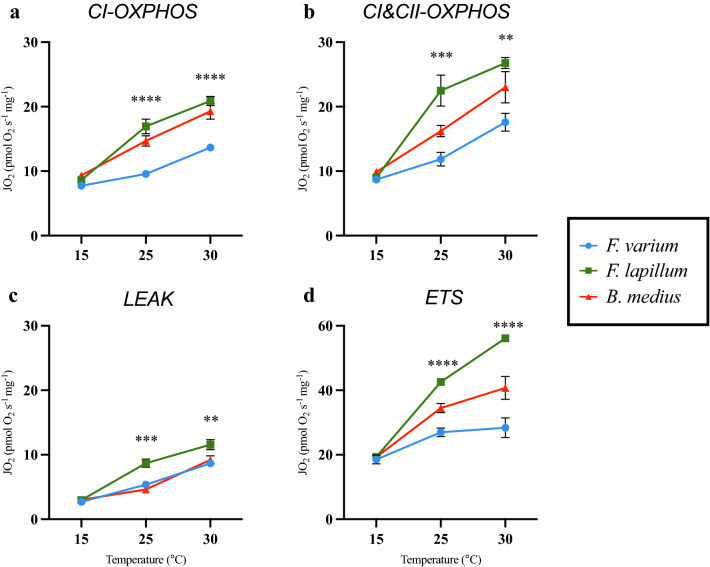
Respiratory flux in each respiration state of mitochondria at 15, 25, and 30 °C. Mean respiratory flux normalized to tissue wet weight (pmol O_2_ s^−1^ mg^−1^) following the modified SUIT protocol for respiration and ATP determination. CI-OXP is initiated by the addition of PMG in the presence of ADP, CI&CII-OXP is initiated by the addition of S. CI&CII-Leak (denoted LEAK) is measured following the addition of the inhibitors Oligomycin (Oli) and carboxyatractyloside (cAtr) while rates of uncoupled respiration (denoted ETS) were measured after complete uncoupling with CCCP. (**a**) Mitochondrial flux during “CI-OXPHOS” for *F. varium, F. lapillum* and *B. medius* across the three experimental temperatures. (**b**) Mitochondrial flux during “CI&CII-OXPHOS” for *F. varium, F. lapillum* and *B. medius* across the three experimental temperatures. (**c**) Mitochondrial flux during “LEAK” for *F. varium, F. lapillum* and *B. medius* across the three experimental temperatures. (**d**) Mitochondrial flux during “ETS” for *F. varium, F. lapillum* and *B. medius* across the three experimental temperatures. Significant differences of p ≤ 0.05 between species within states are denoted by an asterisk (*), significant differences of p ≤ 0.01 are denoted (**), differences of p ≤ 0.001 are denoted (***) while differences of p ≤ 0.0001 are denoted (****).

A SUIT protocol was applied to stimulate mitochondrial respiration in brain mitochondria. The respiratory flux at 15 °C, showed no differences among species for any of the mitochondrial states (Fig. 3). Differences were seen across all states at 25 °C (Fig. 3). Both *F. lapillum* and *B. medius* showed higher flux during CI-OXPHOS and CI&CII-OXPOS compared with *F. varium* (Fig. 3a,b; *B. medius:* CI-OXPHOS, p = 0.007; CI&CII-OXPHOS, p = 0.025; *F. lapillum:* CI-OXPHOS, p = 0.001; CI&CII-OXPHOS, p < 0.0001). ETS rates differed between all three species, with *F. lapillum* having the highest rates compared with *B. medius* and *F. varium* (p < 0.0001). Differences were seen between *F. lapillum* and *B. medius* at CI&CII-OXPHOS (Fig. 3b, 25 °C: p = 0.008) and LEAK (Fig. 3c, 25 °C: p < 0.0001; 30 °C: p = 0.008). ETS rates significantly differed between all species at 30 °C with *F. varium* displaying the lowest rate (p < 0.0001). CI-OXPHOS (p = 0.013) and CI&CII-OXPHOS (p = 0.001) also differed significantly with *F. varium* having the lowest rates compared with *F. lapillum* at 25 °C and 30 °C (Fig. 3a,b).

**Figure 4:**
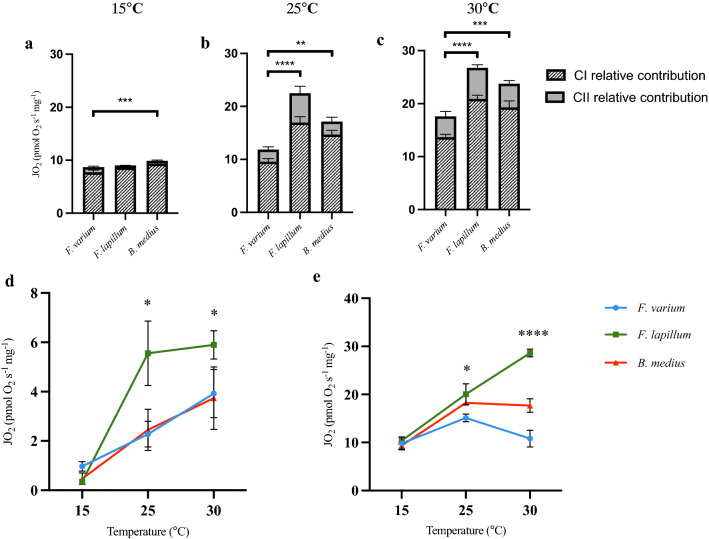
Complex contributions (CI&CII) to OXPHOS and reserve respiratory capacity of mitochondria at 15, 25 and 30 °C. (**a**–**c**) Complex I (CI and complex II (CII) contribution to OXPHOS by temperature (**a**; 15 °C, **b**; 25 °C, **c**; 30 °C). (**d**) Complex II activity contribution to OXPHOS across temperature. (**e**) Respiratory reserve capacity calculated as ETS-CI&CII OXPHOS across temperature. Significant differences between species within temperature of p ≤ 0.05 are denoted by an asterisk (*), significant differences of p ≤ 0.01 are denoted (**) while differences of p ≤ 0.0001 are denoted (****).

Complex II contribution to mitochondrial flux was calculated as CI-OXPHOS subtracted from CI&CII-OXPHOS (Fig. 4a–d). Differences were seen at 25 °C with *F. lapillum* showing greater CII contribution compared with *B. medius* (Fig. 4b,d; 25 °C, p = 0.026) and *F. varium* (Fig. 4b,d; 25 °C, p = 0.018). Reserve respiratory capacity was calculated as CI&CII-OXPHOS subtracted from ETS. This is a measure of excess capacity above CI&CII-OXPHOS available to the mitochondria. Reserve capacity increases for all three species between 15 and 25 °C, with *F. lapillum* having greater capacity compared to *F. varium* (Fig. 4e; 25 °C: p = 0.026). Excess capacity plateaus and decreases for both *B. medius* (Fig. 4e; *F. varium*. p = 0.002; *F. lapillum*, p < 0.0001) and *F. varium* (Fig. 4e; *B. medius*, p = 0.002; *F. lapillum,* p < 0.0001) at 30 °C, while it increases in *F. lapillum* with differences seen across all three species (Fig. 4e; *B. medius* and *F. lapillum,* p < 0.0001).

### ATP assays

**Figure 5:**
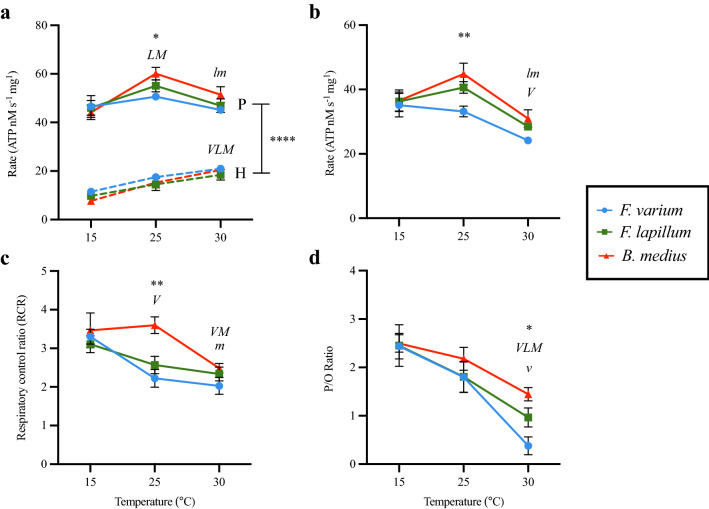
Rates of ATP production and hydrolysis with Respiratory control ratios (RCR) and P/O ratios for all three species at the experimental temperatures (15, 25 and 30 °C). (**a**) ATP production and ATP hydrolysis rates. (**b**) Net ATP production rate. (**c**) RCR’s were calculated as (CI&CII-OXPHOS-LEAK)/CI&CII-OXPHOS and represent the proportion of oxygen consumption coupled to energy production. (**d**) P/O ratios calculated as the rate of overall ATP production divided by O_2_ consumption during CI&CII-OXPHOS. Significant differences between species within temperature of p ≤ 0.05 are denoted by an asterisk (*), significant differences of p ≤ 0.01 are denoted (**) while differences of p ≤ 0.0001 are denoted (****). Significant differences within species across temperature are represented by letters (*v/V* = *F.varium*; *l/L* = *F. Lapillum; m/M* = *B. medius)*. Capitalised letters represent differences from 15 °C while lowercase letters represent differences from 25 °C.

At all temperatures ATP production rates differed substantially from hydrolysis rates for all three species (Fig. 5a; p < 0.0001). At 30 °C overall ATP production rates decline from those at 25 °C for *F. lapillum* and *B. medius* but remain above the increasing ATP hydrolysis rates (Fig. 5a; 30 °C: p < 0.0001). Net ATP production rates decline sharply to closely match hydrolysis rates at 30 °C, and only remained marginally higher for *B. medius* (Fig. 5b; 30 °C: p = 0.011) and *F. lapillum* (Fig. 5b; 30 °C: p = 0.018) but were equivalent for *F. varium* (Fig. 5b; 30 °C: p = 0.8846).

### Respiratory control and P/O ratios

Respiratory control ratios (RCRs) were calculated as (CI&CII-OXPHOS-LEAK)/CI&CII-OXPHOS and these represent the proportion of oxygen consumption commonly assumed to be coupled to ATP synthesis. High RCR values are indicative of tightly coupled mitochondria while low values represent dysfunctional mitochondria. Phosphate/Oxygen or P/O ratios were calculated as the rate of overall ATP production divided by O_2_ consumption during CI&CII-OXPHOS and provide a more accurate, dynamic measure of the amount of ATP produced per molecule of oxygen consumed in the background of ATP hydrolysis. At 25 °C the RCR for *B. medius* was significantly higher than that of *F. varium* (Fig. 5c; p = 0.0067). For P/O ratios, *B. medius* had a greater P/O ratio than that of *F. varium* at 30 °C (Fig. 5d; p = 0.0186). P/O ratios showed a significant decline from 25 to 30 °C in *F. varium* but not for the two other species (Fig. 5d; p = 0.0014).

## Discussion

We demonstrate that brain mitochondrial efficiencies likely play a key role in thermal tolerance and that as temperature increases respiration and ATP production decreases leading to a tight energetic balance. Notably our approach permitted some comparison of ATP synthetic to hydrolysis rates, and the amount of ATP consumed increases as the amount of ATP produced declines. For the subtidal *F. varium* at 30 °C the balance between production and consumption of ATP sits on a knife’s edge (Fig. 5). While the intertidal species, *B. medius* and *F. lapillum*, showed declines in ATP production and consumption as temperature increased, the energetic surplus of ATP synthesis narrows yet remains in excess for these intertidal species at 25 and 30 °C.

### Energetic balance in the brain

The role of neural function in setting the upper thermal limits has been largely ignored. Similar to the heart, the brain is an excitable tissue with high basal energetic demands^23–27^. The changes in mitochondrial efficiency and ATP dynamics experienced during heat stress will mediate dysfunction across the neural system and alter brain function. Early investigations have argued that neural function at the organ and cellular level may limit upper thermal tolerance^11,16,30^. Decreased control of ventilation and blood circulation within the brain was shown as temperature increases^41^, while at the cellular level, action potential conduction and synaptic transmission is sensitive to increasing temperatures in several studies^42–45^. Other recent studies have compared the acclimation responses of heart and brain in eurythermal teleost species. While both organs showed significant acclimation responses, a larger acclimation response was found in the brain^30^. In two crustacean species (*Penaeus monodon* and *Astacus astacus*), the cardiorespiratory system maintained O_2_ supply up to T_crit_ suggesting temperature resistance of the heart; however, the generation and conduction of neuronal action potentials failed approaching T_crit_, suggest a greater thermal sensitivity of the nervous system relative to the heart in these species^12^.

The high energetic demands of the brain require a balance to be maintained between the production of sufficient ATP and its rapid consumption. This study is the first to attempt to formulate a measure of the dynamic energetic balance of ATP production and use, specifically, in terms of the mismatch between energy supply and demand during heat stress. Generation of action potentials involves rapid plasma membrane depolarization and requires repolarization using ATP-dependent ion pumps^46^. With alterations in channel properties, neuronal membranes become leakier with increasing temperature, and active ion repartition becomes more energetically costly^47,48^. Action potentials must also exceed a threshold and increased temperature decreases action potential amplitude and duration, decreasing propagation in excitable tissue^48^. An additional large ATP sink involves the extrusion of neurotransmitters into synaptic clefts to transmit signals, and this increases with heat stress^44,49^.

Here, we determined mitochondrial function and specifically, relatively simple ATP dynamics. These changed with temperature for all three species, and we observed a clear decrease in mitochondrial stability and capacity to meet ATP requirements as temperature increases. While we used brain homogenates, which may not represent in vivo ATP demands, it provides a proxy measurement and system to measure ATP synthesis and hydrolysis under equivalent conditions. Regardless, the subtidal species’ *F. varium* is the most sensitive to temperature and is at a point of no reserve ATP capacity, while the intertidal *B. medius* shows greater resilience to elevated temperatures.

### Coupling of OXPHOS

OXPHOS rates increased with temperature for all species (Fig. 3a,b) and indicates increased O_2_ consumption. Leak rates from all three species also increased significantly from 15 to 30 °C with the greatest proportion of respiration flux resulting from LEAK in *F. varium* at 30 °C (Fig. 3c). These results align with previous work on permeabilized heart^33^ and skeletal muscle fibres^50^ from triplefin fish, where LEAK O_2_ flux also elevates at high temperatures. This supports the view that mitochondrial function and stability declines prior to CT_max_^15,17,33^. Elevated O_2_ demand as OXPHOS efficiency decreases may become increasingly detrimental if sufficient O_2_ cannot be extracted from water. Notably, *F. varium* and *F. lapillum,* have lesser capacities to extract O_2_ than *B. medius* at low O_2_ partial pressures^51^. The synergistic effects of increasing temperature will increase the drain on O_2_ and substrate (e.g., glucose) to maintain nervous function.

We tested the integrity of the ETS complexes, at 15, 25 and 30 °C. Consistent with previous work^33^, ETS rates increased significantly from 15 to 30 °C in all three species (Fig. 3d); however, the greatest increase in flux was seen in the mid tide species *F. lapillum* (Fig. 3d). Reserve respiratory capacity, calculated as the difference between maximal O_2_ consumption (ETS) and basal respiration (CI&CII-OXPHOS) has been employed to provide an estimate of a tissues capacity to cope with increases in ATP demand^52^. Under “normal” conditions, mitochondria are thought to operate at a lesser fraction of their energetic capacities. For cells such as neurons that experience large fluctuations in energy demand on short timescales, the capacity to increase supply to meet demands are essential. The mid-tide species, *F. lapillum,* had the greatest reserve respiratory capacity at 25 and 30 °C compared with both *F. varium* and *B. medius* (Fig. 4e). Theoretically, this greater reserve capacity will provide *F. lapillum* a wider window to defend and buffer ATP supply following s conditions of stress. However, the elevated LEAK rate at both 25 and 30 °C for *F, lapillum* (Fig. 3c) indicate that less of the consumed oxygen is directed towards ATP production. Our measures of ATP synthesis reflect this effect in the contexts of P/O ratios (Fig. 5b).

### ATP dynamics

Oxygen consumption is a traditional proxy or indirect measure of energy expenditure in aerobic organisms, as most ATP production occurs aerobically^53–55^. However, this assumption is compromised if OXPHOS is uncoupled. Only measures of ATP dynamics, or balance, can determine ATP production rates at elevated temperature and provide insight into function under physiological conditions. This study revealed that at 25 °C ATP production rates are elevated for the two intertidal species while it was diminished for *F. varium.* At 30 °C, and all three species showed significantly diminished ATP production rates and elevated ATP hydrolysis rates (Fig. 5). Work performed on the common New Zealand wrasse (*Notolabrus celidotus*) showed that the capacity of isolated cardiac mitochondria to efficiently produce ATP decreased at 25 °C, prior to temperature induced heart failure at 27.5 °C^15,17^. Work with isolated mitochondria from rat cardiomyocytes also revealed diminished ATP production at elevated temperatures and the eventual reversal of the ATP synthase at 43 °C, making the mitochondria a consumer of ATP^17^. Use of Phosphorus-31 NMR (31P-NMR) during acute heat shock measured the rapid decline in ATP at elevated temperatures in *Tetrahymena* ciliate species^56,57^, and the same technique has been used by others and revealed an immediate fall in ATP after brief exposure to sub-lethal heating^26,57^. While the initial ATP levels were recovered after 48 h, a prolonged exposure to heat stress would likely lead to irreversible loss of ATP and the eventual activation of necrotic pathways and death. In *B. medius* and *F. lapillum*, the exposure to elevated temperatures in rock pools occurs over several hours within a day^34^. While these are sub-lethal regarding their respective CT_max_, the decreases in mitochondrial efficiency and ATP production can lead to the irreversible loss of function. Similarly, exposure to moderate heat stress in liver homogenates of juvenile Brown trout (*Salmo trutta*) induced lowered mitochondrial coupling and increased leak rates and the inferred insufficiency to maintain ATP homeostasis may have diminished food intake and suppressed growth^58^. As expected, ATP hydrolysis rates in this study were highest at 30 °C in all species (Fig. 5a). The temperature sensitive *F. varium* had the lowest rates of ATP production relative to ATP hydrolysis and this is reflected in the declining P/O ratios (Fig. 5a,b). However, at 30 °C all three species showed declines in the efficiency of brain homogenates to produce ATP above the ever-increasing hydrolytic rates of ATP. Prior to their respective CT_max_, all species have decreased mitochondrial efficiency to adequately produce ATP, leading to growing imbalance between ATP demand and ATP production.

Mitochondrial efficiency can be defined by the organelles ability to efficiently transfer the free energy released from reducing pathways to ATP production. Traditionally P/O ratios have been used to quantify the number of ATP molecules produced per molecule of O_2_ consumed. Mechanistic P/O ratios have been previously calculated using end-point protocols in respiration assays^55,59^; while the use of MgG fluorescence has allowed the generation of more accurate “active” and “steady- state” P/O ratios. This technique was utilized in recent studies that have provided insight in terms of mitochondrial efficiency with changing temperatures across a range of species^15,17,37,39^. Traditional approaches were unable to produce P/O ratios that were informative about ATP dynamics, whereas another approach^17^ enabled a more dynamic assessment of production and hydrolysis of ATP. Increases in temperature mediated decreases in P/O ratios as mitochondria became less efficient at producing ATP per O_2_ consumed. At 25 °C there was a near 25% decrease in the P/O ratio from 15 °C in *F. varium.* This was further exacerbated at 30 °C where the P/O ratio was further decreased by up to 75% (Fig. 5b). This decline in P/O ratio means more O_2_ is required to sustain ATP production, this also requires a concomitant increase in other metabolic fuels, such as glucose.

The brain is predominantly aerobic, relying on steady supply of O_2_ and glucose to fuel its activity. With limited capacity for anaerobic metabolism, ATP production at elevated temperatures is time restricted and comes at a potentially greater long-term cost for the individuals. Comparing across species, *B. medius* has the greatest brain tissue glycogen stores compared with both *F. lapillum* and *F. varium*^51^. This paired with the higher P/O ratios at elevated temperatures provides *B. medius* with a greater capacity to function at elevated temperatures it may experience during the tidal cycle. Similar declines in P/O ratios have been shown in studies at elevated temperatures^15,17^. The common New Zealand wrasse (*Notolabrus celidotus*) showed decreased ATP production rate by cardiac mitochondria as temperature increased and showed depressed “active” P/O ratios at 32.5 °C^15^. The “steady- state” P/O ratios calculated by Ref.^17^ showed a decline in P/O ratio from 2.5 at 37 °C down to a negative P/O ratio at 43 °C for rodent heart mitochondria. At 25 °C, all three species could maintain sufficiently high P/O ratios while at 30 °C only the rock-pool exclusive *B. medius* was able to sustain a P/O ratio above one. Compared with respiration data, declining P/O ratios are an accurate indication of mitochondrial efficiency and can be used to assess the dynamic shifts of ATP within the cells.

The greater mitochondrial efficiency seen in *B. medius* coupled with recent work^34,51^; showing greater brain glycogen stores and increased O_2_ extractive capacity, compared with *F. lapillum* and *F. varium,* will aid greater survival at elevated temperatures in the rock pool environment but the closely matched production and hydrolysis of ATP at 30 °C will be further exacerbated as temperatures increase.

## Conclusions

Assessment of the respiration rates showed declines in mitochondrial stability and function at elevated temperatures in the subtidal species, *F. varium*, which do not experience high temperature fluctuations. However, the higher intertidal species *B. medius* had greater mitochondrial efficiency and stability at elevated temperatures as was expected. These results agree with the responses from other fish species, which show decreases in mitochondrial function prior to CT_max_ and may be involved in setting upper thermal tolerance limits. Assessment of ATP dynamics in real-time showed that the mitochondrial capacity to produce ATP was diminished at elevated temperatures as ATP hydrolysis rates increased. This led to a closely matched supply and demand dynamic of ATP that may become further exacerbated at CT_max_ and could be involved in underpinning the upper thermal tolerance limit.

## Supplementary Information

Supplementary Figure 1.

## Data Availability

The dataset supporting the results of this manuscript is available from the University of Auckland repository Research Space: https://figshare.com/s/e4d854208b6c8bcc8af7.
