# Supplementary material for: Thermally tolerant intertidal triplefin fish (Tripterygiidae) sustain ATP dynamics better than subtidal species under acute heat stress
Source: Sci Rep. 2021 May 26;11:11074. doi: 10.1038/s41598-021-90575-y (PMC8155050; doi:10.1038/s41598-021-90575-y)
Supplement: Supplementary file 1 — Supplementary Figure 1. [file 41598_2021_90575_MOESM1_ESM.docx]

**Thermally tolerant intertidal triplefin fish (Tripterygiidae) sustain ATP dynamics better than subtidal species under acute heat stress**

**Jaime R. Willis^1, *^, Anthony J. R. Hickey^1^, Jules B. L. Devaux^1^**

*^1^School of Biological Sciences, The University of Auckland, Auckland 1142, New Zealand.*

*^*^Author for correspondence (*[*jwil497@aucklanduni.ac.nz*](mailto:jwil497@aucklanduni.ac.nz)*)*

***Supplementary figures***


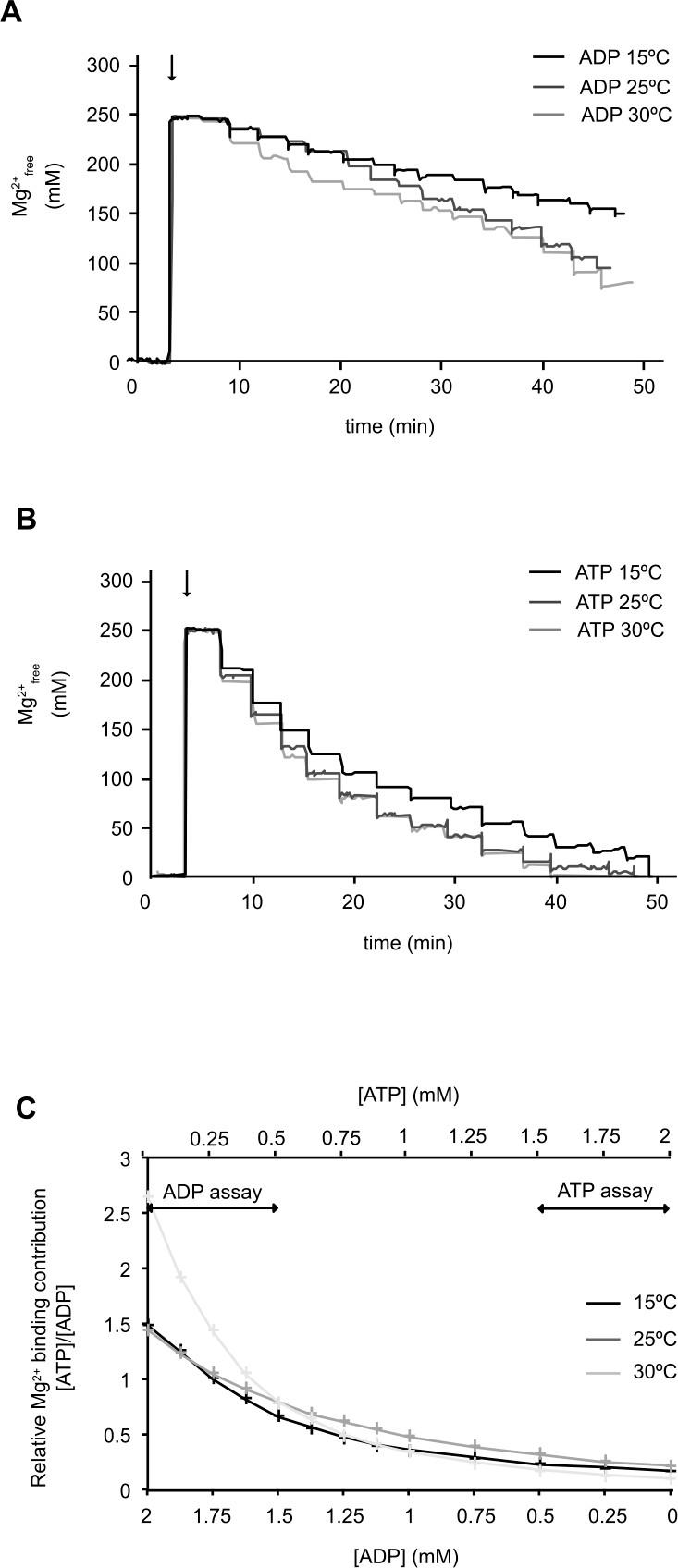


**Supplementary figure 1.** **Calibration assays of the Magnesium Green™ (MgG) fluorescent signal at the three temperatures used for experimental assays.** Separate assays with repeated titrations of ADP **(A)** and ATP **(B)** at 15, 25 and 30°C. MgG (5 mM) was titrated into the assay prior to the MgCl_2_ (2.5 mM)_._ Stepwise titration of either ADP or ATP was made. The steady-state fluorescent signal was reached between each titration. **(C)** The relative binding contributions of ADP and ATP was calculated from **(A)** and **(B)** calibration assays, and covers the concentrations used for the experimental assays (i.e. 1.5-2 mM). This allowed the accurate determination of ATP dynamics.
